# Trends of Enterovirus D68 Concentrations in Wastewater, California, USA, February 2021–April 2023

**DOI:** 10.3201/eid2911.231080

**Published:** 2023-11

**Authors:** Alexandria B. Boehm, Debra A. Wadford, Bridgette Hughes, Dorothea Duong, Alice Chen, Tasha Padilla, Chelsea Wright, Lisa Moua, Teal Bullick, Maria Salas, Christina Morales, Bradley J. White, Carol A. Glaser, Duc J. Vugia, Alexander T. Yu, Marlene K. Wolfe

**Affiliations:** Stanford University, Stanford, California, USA (A.B. Boehm);; California Department of Public Health, Richmond, California, USA (D.A. Wadford, A. Chen, T. Padilla, C. Wright, L. Moua, T. Bullick, M. Salas, C. Morales, C.A. Glaser, D.J. Vugia, A.T. Yu);; Verily Life Sciences, South San Francisco, California, USA (B. Hughes, D. Duong, B.J. White);; Emory University Rollins School of Public Health, Atlanta, Georgia, USA (M.K. Wolfe)

**Keywords:** enterovirus D68, EV-D68, viruses, trends, concentrations, wastewater, acute flaccid myelitis, respiratory infections, zoonoses, California, United States

## Abstract

In this retrospective study, we measured enterovirus D68 (EV-D68) genomic RNA in wastewater solids longitudinally at 2 California, USA, wastewater treatment plants twice per week for 26 months. EV-D68 RNA was undetectable except when concentrations increased from mid-July to mid-December 2022, which coincided with a peak in confirmed EV-D68 cases.

Enterovirus D68 (EV-D68) was recognized as a respiratory virus in 1962 (*1*). In 2014, unprecedented large outbreaks of EV-D68 infections associated with severe respiratory illnesses occurred in children in the United States (*2*), coinciding with a subsequent increase in cases of acute flaccid myelitis (AFM) (*3*). The Centers for Disease Control and Prevention (CDC) began active AFM monitoring in 2014, and as of July 2023, there have been 729 confirmed cases (*4*). Most patients have AFM develop during August‒November. Cyclical peaks in AFM incidence were observed every 2 years (2014, 2016, and 2018) before the COVID-19 pandemic.

CDC began active sentinel surveillance for EV-D68 in 2017 through the New Vaccine Surveillance Network and detected a greater number of EV-D68 cases during July‒August 2022 (*5*). However, there is no formal EV-D68 surveillance at state or local levels. Considering the emergence of wastewater surveillance (WWS) as a method to monitor pathogens at various population levels (*6*), we sought to assess the feasibility of applying WWS to elucidate EV-D68 circulation. WWS could provide an early alert system for public health authorities to mitigate possible increases in severe acute respiratory illness and AFM and to enhance physician awareness during times of increased circulation.

EV-D68 is shed in respiratory secretions and feces (*7*), although data are limited. Two recent studies reported EV-D68 RNA in wastewater in 2021 and concordance with confirmed infections in Israel and the United Kingdom (*8*,*9*). In this retrospective, longitudinal study, we developed an EV-D68 assay, deployed it for WWS, and compared WWS results with laboratory-confirmed EV-D68 cases. This study was reviewed by the State of California Health and Human Services Agency Committee for the Protection of Human Subjects and determined to be exempt from oversight.

## The Study

We tested the EV-D68–specific primers and probe developed by Wylie et al. (*10*) for their sensitivity and specificity in silico and in vitro against virus panels, intact viruses, and cDNA gene blocks (Appendix). In silico testing indicated no cross-reactivity with non–EV-D68 sequences deposited in GenBank, and in vitro testing indicated no cross-reactivity with nontarget viral gRNA. However, the assay did not detect the EV-D68 variant circulating in fall 2022 in the Northern Hemisphere (EV-D68–2022).

We therefore developed a new set of primers, and modified the probe, to amplify and detect in EV-D68–2022 the same region of the polyprotein region (viral protein 1) gene targeted by Wylie et al. We downloaded EV-D68–2022 genome sequences from GenBank in September 2022, aligned them to identify conserved regions in viral protein 1, and developed primers and probes in silico (Appendix Table 1). We confirmed the new primers and probes were specific and sensitive in silico and in vitro. The EV-D68 assay used in this study uses 2 forward and 2 reverse primers to ensure detection of all EV-D68 variants (Table).

**Table:** Forward and reverse primers and probe used in study of trends of EV-D68 concentrations in wastewater, California, USA, February 2021–April 2023*

Target	Primer/probe	Sequence, 5′ → 3′
EV-D68 VP1	Forward	CACYGAACCAGARGAAGCCA and CACTGAACCAGAGGAAGCTA
	Reverse	CCAAAGCTGCTCTACTGAGAAA and CTAAAGCTGCCCTACTAAGRAA
	Probe	TCGCACAGTGATAAATCARCAYGG

*EVD-68, enterovirus D68; VP, viral protein.

We retrospectively selected wastewater solids grab samples from biobanked samples collected as part of a prospective, longitudinal WWS program in California. We selected samples from wastewater treatment plants in San Jose , serving 1.5 million persons in Santa Clara County and Oceanside, serving 250,000 persons in San Francisco County (Appendix Figure 1). We collected and analyzed 2 samples that were collected from each site per week during February 1, 2021‒April 24, 2023.

We thawed wastewater solids overnight and extracted nucleic acids from 10 replicate sample aliquots as described (*11*). We used RNA as template in 10 replicate digital droplet reverse transcription PCR wells for each sample to measure EV-D68 RNA. We measured pepper mild mottle virus RNA as an endogenous control.

Details of reverse transcription PCR chemistry and data processing are provided (Appendix). We report measurements as copies per gram dry weight of solids (copies/g) with SDs. The lower limit of detection was ≈500 copies/g.

During February 2021‒April 2023, EV-D68 RNA concentrations in wastewater solids were not detectable except for samples collected during mid-July 2022 to mid-December 2022. During that time, EV-D68 RNA concentrations increased to ≈10^5^ cp/g in mid-October at both plants (Figure, panels A, B). Plots of EV-D68 normalized by pepper mild mottle virus were similar (Appendix Figure, panels A, B).

**Figure:**
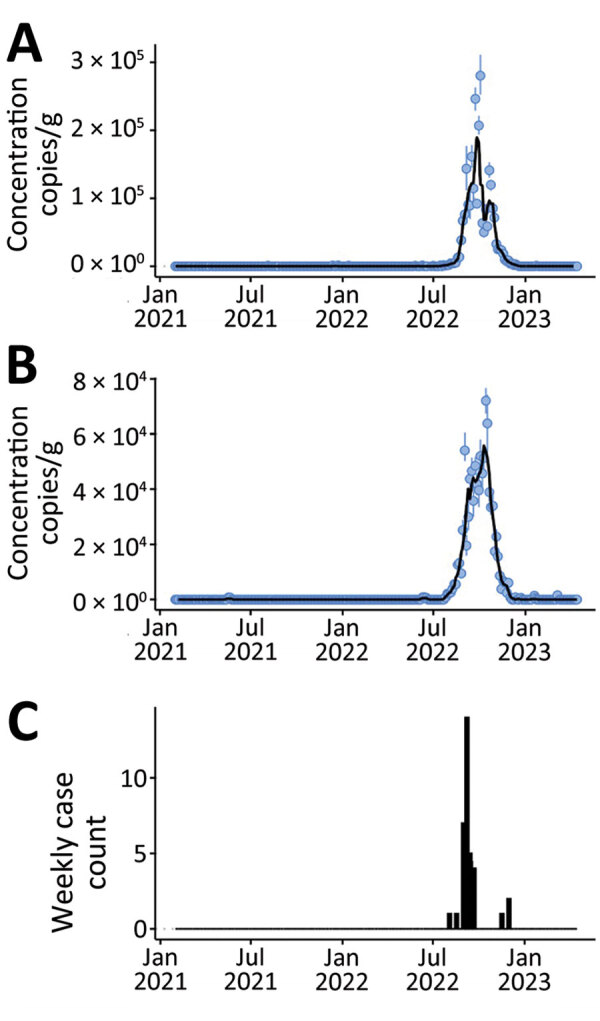
Enterovirus D68 RNA concentrations in wastewater solids from 2 wastewater treatment plants, California, USA. A, B) San Jose plant (A), serving 1.5 million persons in Santa Clara County, and Oceanside plant (B), serving 250,000 persons in San Francisco County. Error bars indicate SDs. Black lines indicate 5-sample trimmed means and are shown for data visualization purposes only. C) Weekly and state-aggregated laboratory-confirmed enterovirus D68 cases in California.

Although there is no active EV-D68 surveillance in California, the California Department of Public Health Viral and Rickettsial Disease Laboratory (VRDL) accepts enterovirus-reactive specimens from hospitals, clinics, and local public health laboratories for strain typing. Enterovirus typing enables us to know, at a limited level, which enterovirus types are circulating and associated with illness. Since 2020, the VRDL has maintained an AFM surveillance program with CDC (*12*). This enhanced AFM surveillance captures patient information but is not specific for EV-D68. However, enterovirus testing and typing are conducted when appropriate sample types are submitted. We aggregated the 35 EV-D68 clinical samples confirmed by VRDL during January 2021–April 2023 by week (Figure, panel C). The average time from date of collection to reporting out EV-D68 result was 32 days (range 12–63 days) (data not shown).

Trends in EV-D68 RNA wastewater concentrations at the 2 California communities match the trend in the state-aggregated weekly data on laboratory-confirmed EV-D68 cases (Figure). Weekly mean wastewater EV-D68 RNA concentrations were positively correlated with weekly case counts for both sites (Kendalls τ = 0.47 for San Jose and 0.50 for Oceanside; p<0.001).

## Conclusions

Detection of EV-D68 in wastewater over 26 months in 2 California communities corresponded strikingly with the trend in statewide laboratory-confirmed EV-D68 cases, although specific EV-D68 case data were not available from those 2 communities (Appendix Table 3). Passive enterovirus surveillance and sentinel EV-D68 surveillance in the United States do not detect most enterovirus cases and focus primarily on the most severe enterovirus cases causing acute respiratory illness, meningitis, and AFM. Most persons infected with enterovirus are either asymptomatic or have mild symptoms and would not likely be tested. For persons who are tested, enterovirus-specific testing is rare, and a presumptive diagnosis is often based on the rhinovirus/enterovirus test on a respiratory virus panel assay without further characterization. Even in cases where EV-D68 is identified, EV-D68 infection is not a reportable disease and not necessarily captured in public health surveillance systems. During July‒December 2022, when EV-D68 RNA was detected and peaked in wastewater, case data were sparse, even when aggregated at the state level (Figure, panel C).

Given that recent EV-D68 surges have been associated with severe pediatric respiratory illness and coincided with increased numbers of AFM cases, there is a benefit to enhancing EV-D68 surveillance so that its circulation dynamics are better understood. Specific testing is rarely done on clinical samples, and surveillance for EV-D68 is limited. When available, EV-D68 typing of clinical cases is seldom timely. Because WWS results are specific for EV-D68 and available 24 hours after sample collection, early warning of EV-D68 levels could be available irrespective of clinical testing. WWS for EV-D68 can inform public health action, including when to issue alerts to improve clinical recognition of the potential for severe respiratory illnesses and AFM cases. Our findings support routine prospective WWS for EV-D68 to inform public health surveillance. However, monitoring EV-D68 target sequences is necessary to ensure appropriate primers and probes are used for EV-D68 detection in wastewater.

Appendix Additional information on trends of enterovirus D68 concentrations in wastewater, California, USA, February 2021–April 2023.
